# A hyperpromiscuous antitoxin protein domain for the neutralization of diverse toxin domains

**DOI:** 10.1073/pnas.2102212119

**Published:** 2022-02-04

**Authors:** Tatsuaki Kurata, Chayan Kumar Saha, Jessica A. Buttress, Toomas Mets, Tetiana Brodiazhenko, Kathryn J. Turnbull, Ololade F. Awoyomi, Sofia Raquel Alves Oliveira, Steffi Jimmy, Karin Ernits, Maxence Delannoy, Karina Persson, Tanel Tenson, Henrik Strahl, Vasili Hauryliuk, Gemma C. Atkinson

**Affiliations:** ^a^Department of Experimental Medicine, University of Lund, 221 84 Lund, Sweden;; ^b^Department of Molecular Biology, Umeå University, 901 87 Umeå, Sweden;; ^c^Center for Bacterial Cell Biology, Biosciences Institute, Newcastle University, Newcastle upon Tyne NE2 4AX, United Kingdom;; ^d^University of Tartu, Institute of Technology, 50411 Tartu, Estonia;; ^e^Department of Clinical Microbiology, Rigshospitalet, 2200 Copenhagen, Denmark;; ^f^Department of Medical Biochemistry and Biophysics, Umeå University, 901 87 Umeå, Sweden;; ^g^Center for Structural Systems Biology, Deutsches Elektronen-Synchrotron, 22607 Hamburg, Germany;; ^h^Department of Chemistry, Umeå University, 901 87 Umeå, Sweden;; ^i^Département Génie Biologique, Campus SophiaTech, Université Nice Sophia Antipolis, 06000 Nice, France;; ^j^Laboratory for Molecular Infection Medicine Sweden, Umeå University, 901 87 Umeå, Sweden

**Keywords:** toxin, antitoxin, bacteriophage, evolution, panacea

## Abstract

Toxin–antitoxin (TA) gene pairs are ubiquitous in microbial chromosomal genomes and plasmids as well as temperate bacteriophages. They act as regulatory switches, with the toxin limiting the growth of bacteria and archaea by compromising diverse essential cellular targets and the antitoxin counteracting the toxic effect. To uncover previously uncharted TA diversity across microbes and bacteriophages, we analyzed the conservation of genomic neighborhoods using our computational tool FlaGs (for flanking genes), which allows high-throughput detection of TA-like operons. Focusing on the widespread but poorly experimentally characterized antitoxin domain DUF4065, our in silico analyses indicated that DUF4065-containing proteins serve as broadly distributed antitoxin components in putative TA-like operons with dozens of different toxic domains with multiple different folds. Given the versatility of DUF4065, we have named the domain *Panacea* (and proteins containing the domain, PanA) after the Greek goddess of universal remedy. We have experimentally validated nine PanA-neutralized TA pairs. While the majority of validated PanA-neutralized toxins act as translation inhibitors or membrane disruptors, a putative nucleotide cyclase toxin from a *Burkholderia* prophage compromises transcription and translation as well as inducing RelA-dependent accumulation of the nucleotide alarmone (p)ppGpp. We find that Panacea-containing antitoxins form a complex with their diverse cognate toxins, characteristic of the direct neutralization mechanisms employed by Type II TA systems. Finally, through directed evolution, we have selected PanA variants that can neutralize noncognate TA toxins, thus experimentally demonstrating the evolutionary plasticity of this hyperpromiscuous antitoxin domain.

## Significance

Toxin–antitoxin systems are enigmatic and diverse elements of bacterial and bacteriophage genomes. We have uncovered remarkable versatility in an antitoxin protein domain that has evolved to neutralize dozens of different toxin domains. We find that antitoxins carrying this domain—Panacea—form complexes with their cognate toxins, indicating a direct neutralization mechanism, and that Panacea can be evolved to neutralize a noncognate and nonhomologous toxin with just two amino acid substitutions. This raises the possibility that this domain could be an adaptable universal or semi-universal protein neutralizer with significant biotechnological and medical potential.

Toxin–antitoxin systems (TAs) are diverse two-gene elements that are widespread in plasmids and chromosomes of bacteria and archaea (1, 2) as well as in genomes of temperate bacteriophages that prey on these microbes (3–6). The various protein toxins target different core processes of the encoding cell to dramatically inhibit growth while their cognate antitoxins efficiently neutralize the toxicity. Known TA toxins exert their toxicity in a variety of ways (1), often targeting translation through modification or cleavage of the ribosome, translation factors, transfer RNAs (tRNAs), or messenger RNAs (mRNAs). Similarly, antitoxins counteract the toxins through diverse mechanisms (1, 7). The means of neutralization are often classified into four or more subtypes, the main four being base pairing of the antitoxin RNA with the toxin mRNA (Type I TA systems), direct protein–protein binding and inhibition (Type II), inhibition of the protein toxin by the antitoxin RNA (Type III), or indirect nullification of toxicity (Type IV). Plasmid-encoded TA systems have long been known to function as addiction modules that promote plasmid maintenance and stability (8). The biological function of chromosomal TAs has been harder to pin down, with different TAs being implicated—with varying levels of confidence—in modulation of bacterial physiology in response to the environment, stabilization of genomic elements, and bacteriophage defense (1, 2, 9, 10).

We have recently discovered a class of TA systems that employs RelA/SpoT homologue (RSH) enzymes—so-called toxic Small Alarmone Synthetases (toxSASs)—as toxic enzymes to abrogate bacterial growth (4). Housekeeping RSH enzymes such as the *Escherichia coli* ribosome-associated amino acid starvation sensor RelA synthesize the nucleotide alarmone (p)ppGpp, a pyrophosphorylated derivative of GDP/GTP (11, 12). The toxicity of *Cellulomonas marina* toxSAS FaRel relies on the production of the related toxic alarmone (pp)pApp from housekeeping adenosine nucleotides AMP, ADP, and ATP (4). The accumulation of (pp)pApp results in dramatic depletion of ATP, which, in turn, leads to the cessation of transcription followed by the inhibition of translation and replication (4, 13). The synthesis of (pp)pApp is not the only mechanism of toxicity employed by toxSAS enzymes: we have found that the majority of experimentally explored toxSASs, such as PhRel2 from *Bacillus subtilis* strain Ia1a, act as specific protein synthesis inhibitors that pyrophosphorylate the 3′ CCA end of tRNA to abrogate aminoacylation (12). In the case of Mycobacterial phage Phrann protein Gp29, a translation-inhibiting toxSAS in the PhRel subfamily that likely pyrophosphorylates tRNA (12), the biological function appears to be defense against phage superinfection (5).

ToxSASs are neutralized by several different antitoxins that act via Type II and Type IV mechanisms. The cognate antitoxin of *B. subtilis* Ia1a PhRel2 (a tRNA-modifying toxSAS) belongs to a widespread domain family of unknown function designated by the Pfam database as DUF4065, in which DUF stands for domain of unknown function (14). Clues about the roles of DUF4065 are limited; however, it is found in so-called genetic element protein A, previously associated with TA loci (15, 16), and is also present in the proteolysis-promoting SocA antitoxin of the replication-inhibiting SocB toxin (17). This unusual mechanism of neutralization by an antitoxin is referred to as Type VI. We have earlier identified the DUF4065 domain in a putative alternative antitoxin to the ribonuclease (RNase) MqsR, but this was not tested experimentally (15).

We asked whether, given the broad distribution of DUF4065 across multiple phyla of bacteria and archaea, the analysis of the genomic neighborhood of DUF4065 can enable the prediction of novel TA systems. Using our tool FlaGs (for flanking genes) (18) to analyze diverse genomes across the tree of life, we find that DUF4065 is the predicted antitoxin counterpart of at least 1,268 different putative TA system families corresponding to at least 88 distinct putative toxin–DUF4065 domain combinations, found in diverse bacteria, archaea, and bacteriophages. While many of the toxins of these systems are related to classical TA toxins such as various mRNA interferases (19, 20), Fic/Doc-type protein modification enzymes (21), and toxSASs (4), others have little similarity to known domains or proteins with solved structures. We have experimentally verified nine DUF4065-containing antitoxins as neutralizers of their cognate toxin partners. These toxins include translation inhibitors, membrane disruptors, and a putative nucleotide cyclase that pleiotropically affects metabolism, compromising transcription and translation, as well as inducing RelA-dependent accumulation of the guanosine tetraphosphate alarmone nucleotide (p)ppGpp. Complex formation indicates DUF4065-containing antitoxins neutralize toxins via direct protein–protein interaction [that is, act as Type II TA systems (1, 2)], and we have identified substitutions that confer the ability of one antitoxin to neutralize a noncognate toxin. Given the versatility of the antitoxin function of DUF4065, we have named the domain *Panacea* after the Greek goddess of universal remedy.

## Results

### The Domain DUF4065 Is Found in Diverse TA-Like Loci across Bacteria, Archaea, and Bacteriophages.

As DUF4065 has previously been associated with TA systems (15–17), we asked whether it may constitute a widespread antitoxin domain paired in operons with novel toxin domains. To answer this, we used sensitive sequence searching combined with an analysis of gene neighborhoods using our tool FlaGs (18) (see *SI Appendix*, Fig. S1 for a graphical overview of the procedure). Using the hidden Markov model (HMM) of the DUF4065 domain (14) to scan 20,209 genomes across cellular life and viruses, we identified 2,281 hits (Dataset S1) in prokaryotes and bacteriophages comprising 27 phyla of bacteria, 3 phyla of archaea, and 17 different bacteriophages (Dataset S1). Of those 2,281, 76 are present in complete prokaryotic genomes, allowing the determination of whether they are chromosome or plasmid encoded according to the genome annotations. All but two of our identified DUF4065 homologs in complete genomes are chromosome localized. The two exceptions annotated as plasmid encoded (but may be minichromosomes) are archaeal, found in Haloarchaea (protein accessions WP_050049451.1 and WP_049938427.1). Most DUF4065-carrying taxa only carry a single homolog; 217 taxa have two, 45 have three, 14 have four, 12 have five, and 5 have more than five. Of these five taxa, the taxon with the most DUF4065 homologs is the Mollicute bacterium “strawberry lethal yellows phytoplasma” strain NZSb11. This genome contains 25 DUF4065 homologs, of which three are predicted as being encoded in TA-like loci by our in silico analysis pipeline.

**Fig. 1.**
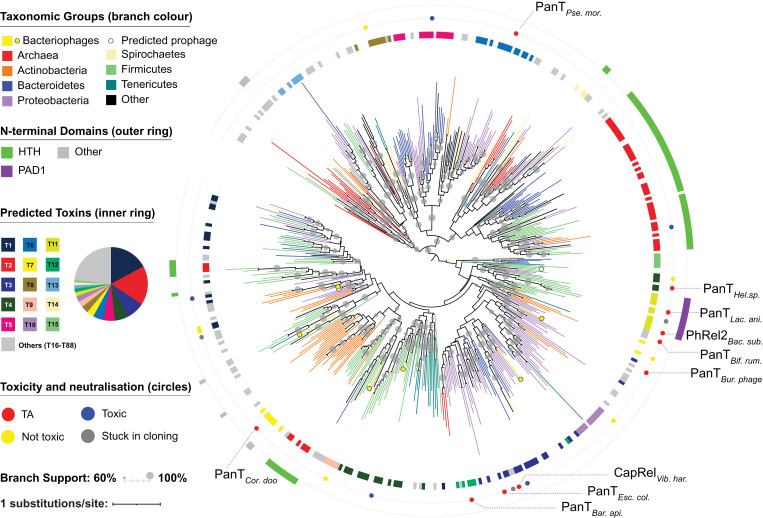
The domain DUF4065/Panacea is found in a wide variety of TA-like loci across bacteria, archaea, and bacteriophages. Branches of the IQTree maximum likelihood phylogenetic tree of representative PanA sequences are colored by major taxonomic groupings as per the upper left key with an additional symbol to highlight bacteriophages. Rectangles in the outer and inner rings indicate the presence and absence of N-terminal domains in the PanA sequences and predicted associated toxin groups, respectively, according to the left-hand keys. Colored circles between the rings indicate putative TA pairs that have been tested in toxicity neutralization assays and the results of those assays. “TA” means the expression of the toxin compromises *E. coli* growth, and coexpression of the antitoxin either fully or partially counteracts the toxicity. “Toxic” means toxicity is confirmed, but the cognate PanA sequence does not rescue in this *E. coli* system. “Stuck in cloning” refers to cases in which the putative toxin genes could not be successfully chemically synthesized and plasmid subcloned, potentially because of the toxicity being too severe. Gray circles on the branches indicate branch support from IQTree ultrafast bootstrapping (53). Tree annotation was carried out with iTOL (54).

Adapting FlaGs for analyzing gene neighborhood conservation (*SI Appendix*, Fig. S1), we find that around half of the identified DUF4065-containing proteins can be detected as being encoded in two-gene loci that are conserved across multiple species, reminiscent of TA systems (Dataset S1, representatives in Fig. 1, Dataset S2, and *SI Appendix*, Fig. S2). In total, we predicted 1,313 preliminarily TA (pTA)-like loci using the criteria 1) that there should be a maximum distance of 100 nucleotides between the two genes, 2) that this architecture is conserved in two or more species, and 3) the conservation of the gene neighborhood does not suggest longer operons than three genes (*SI Appendix*, Fig. S1). We allowed three-gene architectures into our analysis, as TAs can sometimes be found with a conserved third gene, such as *mazG* in the case of *mazEF* (22), chaperones in the case of tripartite TA–chaperone modules (23), or transcriptional regulators in the case of the *paaR-paaA-parE* system (3). By allowing three-part clusters, we have identified 25 clusters that are conserved as a third gene in a subset of genomes that encode a particular predicted TA pair (Dataset S1). We call these accessory proteins, annotations of which include DNA/nucleotide and protein/amino acid modification enzymes, helicases, proteases, and nucleases. Each detected accessory third gene was only present in a small fraction of the genomes in which the main TA pair was identified, suggesting that whatever the role of these third genes, they probably do not play a general role in toxicity and neutralization.

It is conceivable that some homologous genes are found adjacent to DUF4065-encoding genes in multiple genomes purely by chance and are not part of genuine TA systems. Therefore, we used a BlastP-based (24) reciprocity test to filter out putative “toxins” that are at risk as being spurious hits (*SI Appendix*, Fig. S1*C*). From the 1,313 pTA-like loci, we determined that 67 proteins (of which 39 are predicted toxins and 28 are accessory proteins) are likely spurious hits (Dataset S1). Major classes of these spurious hits are transposases/integrases that are commonly found in TA-encoding neighborhoods and various ATPases that are captured into homologous clusters because of their well-conserved ATP-binding motifs (Dataset S1).

The remaining 1,268 putative TA loci that we predict to be relatively reliable correspond to 88 clusters of potential toxins. We number these clusters with a T prefix; for example, SocB is in cluster T10. The vast majority of these are annotated as “hypothetical protein,” as they share only weak similarity to proteins of known function. Therefore, we searched the putative toxin protein sequences against the National Center for Biotechnology Information Conserved Domains Database (NCBI CDD) to detect the presence of known domains (Dataset S1). Of the 1,268 putative toxins, 938 sequences (belonging to 41 clusters) had no hit to a domain, and of the others, the most predominant domains were MqsR like (*n* = 90), Fic/Doc like (*n* = 32), and toxSAS like (domain names NT_Pol-beta-like, RelA_SpoT, and NT_Rel-Spo-like; *n* = 31). Other known toxin domains that were represented in the CDD results were PemK (mRNAse) and ParE (DNA gyrase inhibitor). For clusters that failed to find a hit in the CDD database, HHPred (25) was run with one to two representative sequences per cluster, revealing additional potential homology to proteins of known structures for 30 clusters (Dataset S1; see the following sections of *Results* for examples among our verified TAs).

The variety in the potential toxin domains suggests that the DUF4065 domain may be a universal or semi-universal antitoxin domain capable of neutralizing various different toxic proteins. In light of this, we suggest renaming DUF4065 to Panacea and abbreviate each Panacea-containing putative antitoxin and putative toxin protein as PanA and PanT, respectively. We refer to the two-gene system with the handle PanAT. In each PanAT system, the order of two genes can differ: either antitoxin first or toxin first. However, antitoxin first is the more common arrangement (943 versus 325) as is typical for Type II TA systems (1, 2).

**Fig. 2.**
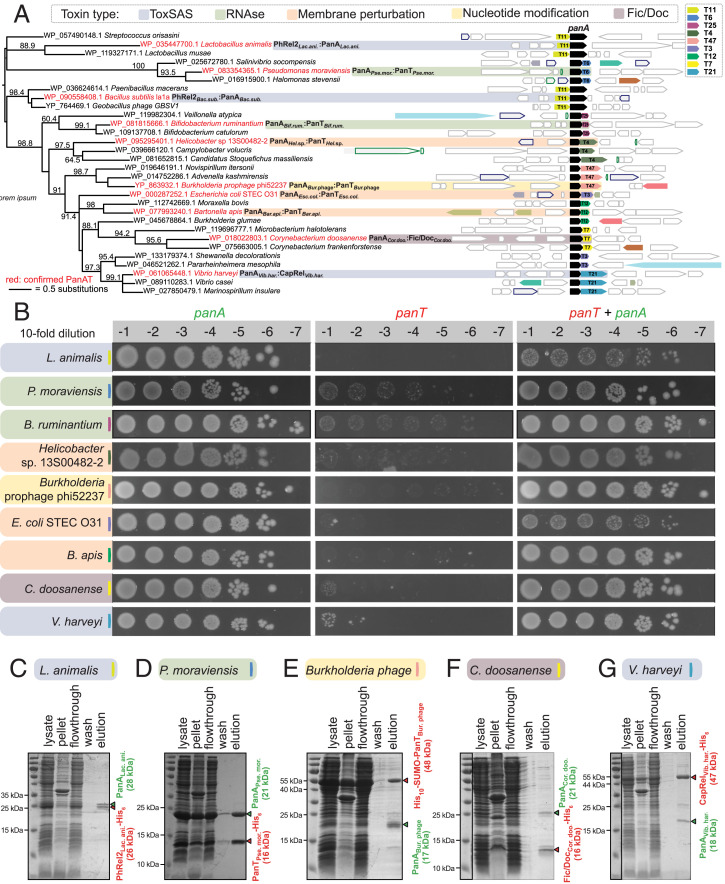
PanA antitoxins form stable complexes with evolutionarily diverse TA toxins. (*A*) The maximum likelihood tree of PanA sequences, annotated with conserved gene neighborhoods generated with FlaGs (18). Numbers on branches show IQTree ultrafast bootstrap support (53). Genes belonging to homologous clusters are colored the same; the PanA antitoxin is universally shown in black. Numbers on genes preceded by a T indicate toxin clusters. (*B*) Validation of *panAT* TA pairs by toxicity neutralization assays. Overnight cultures of *E. coli* strains transformed with pBAD33 and pKK223-3 vectors or derivatives expressing putative *panT* toxins and *panA* antitoxins, correspondingly, were adjusted to OD_600_ 1.0, serially diluted, and spotted on LB medium supplemented with appropriate antibiotics and inducers (0.2% arabinose for *panT* induction and 1 mM IPTG for *panA* induction). (*C*–*G*) A pull-down assay demonstrates complex formation between PanA antitoxins and PanT toxins. Untagged PanA representatives were coexpressed in the *E. coli* BL21 DE3 strain together with N-terminally affinity-tagged (His_10_-SUMO in the case of *Burkholderia* prophage phi52237 PanT; His_6_ in all other cases) cognate PanT toxin. Filtered lysate was incubated with buffer-equilibrated Ni-beads, and PanAT complexes were eluted with 300 mM imidazole and resolved on 15% SDS-PAGE. The theoretical molecular weights for tagged toxins and antitoxins are indicated in red and green, respectively.

Maximum likelihood phylogenetic analysis shows the PanA tree largely does not follow taxonomic relationships, reflecting a high degree of mobility (Fig. 1, *SI Appendix*, Fig. S2, and Dataset S2). While the deepest branches are poorly supported (not surprising for a small protein), there are a number of groups with medium to strong (over 60 to 100%) bootstrap support that include different bacterial—and sometimes archaeal—phyla. While Panacea is present broadly across prokaryotes, it does not appear to be present in eukaryotes. The only PanA we discovered in eukaryotes was in the Pharoah ant (*Monomorium pharaonic*; XP_028045404.1), and this appears to be a case of contamination, as an identical sequence is found in the bacterium *Stenotrophomonas maltophilia*. Surprisingly, a strongly supported clade of PanA sequences does not necessarily mean they all share the same PanT as shown by the inner ring in Fig. 1 and the toxin partner swapping in focus in Fig. 2*A* and *SI Appendix*, Fig. S2. Indeed, the exchange of toxin partners within a clade appears to be frequent. We refer to this kind of domain-level partner swapping as *hyperpromiscuity*, to distinguish from the promiscuity that can be seen when one single antitoxin sequence can nullify multiple con-cognate but homologous toxins (26–28).

Some—but not all—PanAs carry additional N-terminal domain regions (Fig. 1). Often, these match a known helix-turn-helix (HTH) domain, of which a number of variations exist in the NCBI CDD. We aligned all the identified regions with hits to HTH models to make our own updated HTH model. From this, we identified HTH domains in the N-terminal regions of 343 PanA sequences (Dataset S1). HTH domains are often DNA binding, are frequently found in transcription factors, and are common in Type II antitoxins (1, 2). This suggests that, like many other Type II systems, Panacea domain–containing antitoxins can in some cases also regulate the TA function at the level of transcription. Apart from HTH domains, the only widely conserved N-terminal extension appears to correspond to a new domain, which we refer to as PanA-associated domain 1 (PAD1) (*SI Appendix*, Fig. S3). All but two of the TA-predicted PAD1 containing PanAs are paired with toxSAS-like toxins (the exception being putative ATPases from Clostridia [PanT group T62; Dataset S1]). The position at the N terminus and the presence of conserved histidines may indicate that PAD1 is a new DNA-binding domain, although it has no detectable homology with any known domain. PAD1 is also present in nine Panacea-containing proteins that do not meet the criteria for TA-like loci (Dataset S1). In all cases in which PanA contains the PAD1 domain and is in a TA-like locus, the toxin is encoded upstream of the antitoxin, the less common arrangement in the data set as a whole and in TA systems in general (1, 2).

### PanA Is a Hyperpromiscuous Antitoxin Domain.

Sampling broadly across PanA diversity, we selected 25 of the putative novel TAs for experimental validation in toxicity neutralization assays (Figs. 1 and 2*A*, Table 1, *SI Appendix*, Table S1, and Dataset S2). Putative toxins and antitoxins were expressed in *E. coli* strain BW25113 under the control of arabinose- and isopropyl β-D-1-thiogalactopyranoside (IPTG)-inducible promoters, respectively (4). For a gene pair to classify as a bona fide TA, two criteria need to be fulfilled: 1) the expression of the toxin should compromise *E. coli* growth, and 2) coexpression with the antitoxin should—either fully or partially—rescue from growth inhibition by the toxin. In addition to the PanA-neutralized PhRel2*_Bac. sub._* toxSAS from *B. subtilis* Ia1a that we have validated earlier (4), we have verified here nine PanAT pairs as being genuine TA loci (Table 1 and Fig. 2*B*).

**Table 1.** Summary of experimentally characterized PanAT pairs

Organism	Description	Toxin MOA/toxin family	Toxin accession	Antitoxin accession
*Escherichia coli* STEC O31	PanA*_Esc. col._*-PanT*_Esc. col._*	Membrane	WP_000019185.1	WP_000287252.1
*Helicobacter* sp. 13S00482-2	PanA*_Hel._*_sp__._-PanT*_Hel._*_sp._	Membrane	WP_095295403.1	WP_095295401.1
*Bartonella apis*	PanA*_Bar. api._*-PanT*_Bar. api._*	Membrane	WP_077993242.1	WP_077993240.1
*Burkholderia* prophage phi52237	PanA*_Bur._*_phage_-PanT*_Bur._*_phage_	Nucleotide cyclase	YP_293707.1	YP_863932.1
*Bifidobacterium ruminantium*	PanA*_Bif. rum._*-PanT*_Bif. rum._*	RNase	WP_026646888.1	WP_081815666.1
*Pseudomonas moraviensis*	PanA*_Pse. mor._*-PanT*_Pse. mor._*	RNase	WP_083201923.1	WP_083354365.1^†^
*Vibrio harveyi*	PanA*_Vib. har._*-CapRel*_Vib. har._*	toxSAS	WP_061065447.1	WP_061065448.1
*Bacillus subtilis* Ia1a	PanA*_Bac. sub._*-PhRel2*_Bac. sub._*	toxSAS	WP_090558406.1	WP_090558408.1
*Lactobacillus animalis*	PanA*_Lac. ani._*-PanT*_Lac. ani._*	toxSAS	WP_052006344.1	WP_035447700.1
*Corynebacterium doosanense*	PanA*_Cor. doo._*-PanT*_Cor. doo._*	Fic/Doc	WP_018022804.1	WP_018022803.1

^†^The NBCI sequence of PanA from *P. moraviensis* appeared to be truncated at the N terminus relative to its homologues, and therefore we took an upstream start codon, equivalent to adding ten amino acids, MIFSEQKVAQ, to the N terminus.

PanA-neutralized toxins from *Lactobacillus animalis* (PhRel2*_Lac. ani._*) and *Vibrio harveyi* (CapRel*_Vib. har._*) belong to two different toxSAS subfamilies and, as we have shown recently, the majority of toxSAS target translation by inhibiting tRNA aminoacylation through the pyrophosphorylation of the 3′ CCA end of tRNA (12). Toxins from *Pseudomonas moraviensis* strain LMG 24280 (PanT*_Pse. mor._*) and *Bifidobacterium ruminantium* strain DSM 6489 (PanT*_Bif. rum._*) have no hits against the NCBI CDD but are predicted to be structurally similar to EndoA/PemK/MazF family RNases with HHPred (25) and thus may act as translational inhibitors similarly to the archetypal TA toxin MazF that cleaves mRNA at ACA nucleotide sequences (29). The *Corynebacterium doosanense* toxin (PanT*_Cor. doo._*) is predicted to be a member of the Fic/Doc protein family, which includes the Doc TA toxin that inhibits protein synthesis by phosphorylating the essential translation elongation factor EF-Tu (21). *Burkholderia* prophage phi52237 (PanT*_Bur._*_phage_) has no detectable homology to any protein domain in the NCBI CDD. However, HHpred predicts similarity to adenylate and guanylate cyclase with 97% probability, suggesting that its toxicity could be via the production of a toxic cyclic nucleotide species. Finally, many of the predicted toxin genes encode putative small peptides with predicted transmembrane helices (*SI Appendix*, Fig. S4). Of the verified TAs, the toxins with putative membrane spanning segments are those originating from *E. coli* strain STEC O31 (PanT*_Esc. col._*), *Helicobacter* sp. 13S00482-2 (PanT*_Hel._*_sp._), and *Bartonella apis* strain BBC0122 (PanT*_Bar. api._*) (Table 1 and *SI Appendix*, Fig. S4). The clusters containing PanT*_Esc. col._* (T3) and PanT*_Bar. api._* (T12) have similar sequence compositions consisting of a charged N-terminal region followed by a hydrophilic C-terminal region where the transmembrane regions are predicted (*SI Appendix*, Figs. S2*A* and S4). It is possible that T3 and T12 are homologous, although they are dissimilar enough that they are not clustered together by FlaGs (*SI Appendix*, Fig. S2*A*). The transmembrane helices of PanT*_Hel._*_sp._ are found at its C terminus, while its N-terminal region is similar to coiled-coil regions found in the synaptonemal complex protein 1 superfamily (30) and a *Salmonella* phage tail needle protein (25). For additional confirmation that PanTs vary substantially on the protein fold level, we de novo–predicted the structures of representative validated PanTs*_._* using trRosetta, a deep learning–based method (31) (*SI Appendix*, Fig. S5). In agreement with the HHpred results, the PanTs are predicted to adopt different, structurally unrelated folds.

Of the potential TA pairs that were selected and could not be verified, three of the putative toxin genes could not be successfully plasmid subcloned by the commercial provider (*SI Appendix*, Table S1). While we cannot be sure of the reason for this, it is likely that their toxicity was too severe to allow cloning in *E. coli*. Five PanTs were toxic but were not rescued by their cognate PanA, and in three of these cases, PanA itself was toxic (*SI Appendix*, Table S1 and Fig. S6 *A*–*D*). For example, while the PanA-associated mRNAse MqsR from *Herbaspirillum frisingense* GSF30 was—as we predicted earlier (15)—toxic, its toxicity was not countered by its cognate PanA when coexpressed in *E. coli* (*SI Appendix*, Fig. S6*C*). Finally, eight PanTs were not toxic when tested in *E. coli*—but this does not rule out the possibility of toxicity in the original host (*SI Appendix*, Table S1).

### PanAT Pairs Are Type II TA Systems.

The Panacea domain–containing SocA antitoxin of *Caulobacter crescentus* acts as a proteolytic adaptor, bringing the toxin SocB into contact with the protease ClpXP (17). To test whether other PanAs act as such adapters, we repeated our neutralization assays in *E. coli* strains lacking ClpXP and Lon proteases. These proteases are not necessary for neutralization by PanA (*SI Appendix*, Fig. S7). We therefore hypothesized that the general neutralization mechanism of PanA is through direct binding and inhibition typical of classical Type II systems. To test this, we carried out pull-down assays using coexpressed cognate native PanA antitoxins together with N-terminally affinity-tagged PanT toxins (with either His_6_ or His_10_-SUMO tags). We validated stable complex formation for five PanAT pairs: *L. animalis* PhRel2*_Lac. ani._*:PanA*_Lac. ani._* (Fig. 2*C*), *P. moraviensis* PanT*_Pse. mor._*:PanA*_Pse. mor._* (Fig. 2*D*), *Burkholderia* prophage phi52237 PanT*_Bur._*_phage_: PanA*_Bur._*_phage_ (Fig. 2*E*), *C. doosanense* Fic/Doc*_Cor. doo._*: PanA*_Cor. doo._* (Fig. 2*F*), and *V. harveyi* CapRel*_Vib. har._*:PanA*_Vib. har._* (Fig. 2*G*).

### Protein Synthesis Is a Major Target of PanT Toxins.

**Fig. 3.**
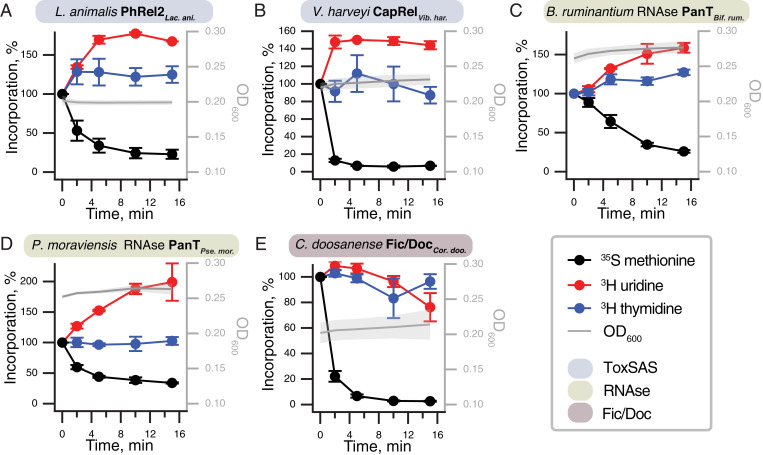
Protein synthesis is a major target of PanT toxins. Metabolic-labeling assays following the incorporation of ^35^S methionine (black traces), ^3^H uridine (red), and ^3^H thymidine (blue) upon expression of translation-inhibiting PanT representatives: (*A*) *L. animalis* PhRel2 and (*B*) *V. harveyi* CapRel toxSAS; putative RNases (*C*) PanT*_Bif. rum._* and (*D*) PanT*_Pse. mor._*; and (*E*) *C. doosanense* Fic/Doc toxin, Fic/Doc*_Cor. doo_*. Expression of PanTs in *E. coli* BW25113 was induced with 0.2% L-arabinose.

To address the molecular mechanisms of PanT toxicity, we assayed the effects of PanT expression on macromolecular synthesis by following the incorporation of ^35^S methionine in proteins, ^3^H uridine in RNA, and ^3^H thymidine in DNA, comparing to the effects of *E. coli* MazF RNase as a positive control (*SI Appendix*, Fig. S8*A*). As predicted, five of the identified PanT—*L. animalis* PhRel2*_Lac. ani._* and *V. harveyi* CapRel*_Vib. har._* toxSAS, putative RNases PanT*_Pse. mor._* and PanT*_Bif. rum._* and *C. doosanense* Fic/Doc toxin, Fic/Doc*_Cor. doo._*—specifically inhibit protein synthesis (Fig. 3 *A*–*E*). The mechanism of action of all the protein synthesis–inhibiting toxins can be predicted by homology. ToxSASs *L. animalis* PhRel2 and *V. harveyi* CapRel are closely related to other representatives we have characterized earlier (12) and almost certainly pyrophosphorylate the CCA end of tRNA. The *C. doosanense* Fic/Doc toxin Fic/Doc*_Cor. doo._* presumably modifies EF-Tu as observed for other Doc enzymes (32). Predicted RNases PanT*_Bif. rum._* and PanT*_Pse. mor._* likely inhibit translation by cleaving mRNA or tRNA as do their—albeit distant—relatives (33).

### *Burkholderia* Prophage phi52237 PanT Is a Pleiotropic Toxin that Induces the RelA-Mediated Stringent Response.

**Fig. 4.**
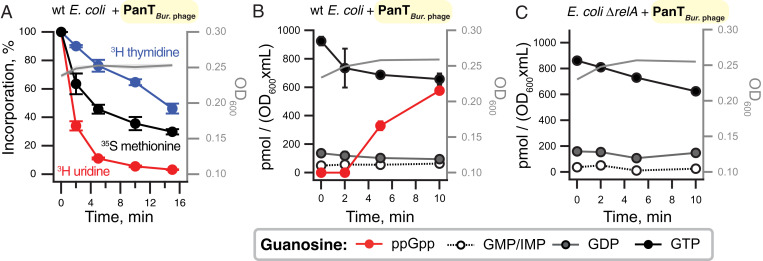
PanT*_Bur._*_phage_ toxin from *Burkholderia* prophage phi52237 compromises transcription and translation as well as inducing the RelA-mediated stringent response. (*A*) Metabolic-labeling assay using wild-type *E. coli* BW25113 expressing PanT*_Bur._*_phage_ toxin. (*B* and *C*) Guanosine nucleotide pools in either wild-type (*B*) or Δ*relA* (*C*) *E. coli* BW25113 expressing PanT*_Bur._*_phage_ toxin. Cell cultures were grown in defined minimal MOPS medium supplemented with 0.5% glycerol at 37 °C with vigorous aeration. Expression of PanT*_Bur._*_phage_ toxin was induced with 0.2% L-arabinose at the OD_600_ 0.2. Intracellular nucleotides are expressed in pmol per OD_600_ × mL as per *inset*. Error bars indicate the SE of the arithmetic mean of three biological replicates.

The *Burkholderia* prophage PanT*_Bur._*_phage_ toxin is unique among our verified toxins in that it predominantly inhibits transcription, with weaker effects on translation and even weaker on replication (Fig. 4*A*). The mode of inhibition is reminiscent of that of *C. marina* FaRel toxSAS (4) and *P. aeruginosa* Type VI secretion system RSH effector Tas1 (12, 13) that act though production of the toxic nucleotide alarmone (pp)pApp, leading to dramatic depletion of ATP and GTP. Therefore, we used our high performance liquid chromatography (HPLC)-based approach to study the effects of PanT*_Bur._*_phage_ toxin expression on *E. coli* nucleotide pools (34). In contrast to the drastic drop in GTP and ATP seen upon expression of *C. marina* FaRel toxSAS (4), expression of PanT*_Bur._*_phage_ results in only a slight decrease in GTP (Fig. 4*B*) without affecting the ATP levels (*SI Appendix*, Fig. S9*A*). Surprisingly, despite having no detectable sequence or structural homology with RSH protein family members, PanT*_Bur._*_phage_ expression causes an accumulation of the alarmone nucleotide ppGpp (Fig. 4*B*). This suggests that either 1) the toxin activates cellular RSH enzymes—given the strength of the effect, likely the most potent *E. coli* (p)ppGpp synthetase RelA—or 2) the PanT*_Bur._*_phage_ toxin itself is capable of producing the alarmone. To distinguish between the two scenarios, we analyzed nucleotide levels upon toxin expression in an *E. coli* strain lacking *relA*. No accumulation of ppGpp is detected upon PanT*_Bur._*_phage_ expression in the *relA*-deficient strain (Fig. 4*C*), and just as in the case of wild type, there is no effect on ATP levels (*SI Appendix*, Fig. S9*B*). Therefore, we conclude that expression of this toxin directly or indirectly induces ppGpp production by RelA. To deconvolute the direct effects of *Burkholderia* prophage PanT*_Bur._*_phage_ toxin on ^35^S methionine, ^3^H uridine, and ^3^H thymidine incorporation from the secondary effects caused by RelA-dependent ppGpp accumulation, we performed metabolic labeling in the Δ*relA E. coli* strain (*SI Appendix*, Fig. S9*C*). Just as in the wild-type strain, the main target is transcription, closely followed by translation. Thus, the growth inhibition and metabolic-labeling effects observed upon PanT*_Bur._*_phage_ expression are not related to ppGpp accumulation.

### The Cell Membrane Is Another Major Target of PanT Toxins.

**Fig. 5.**
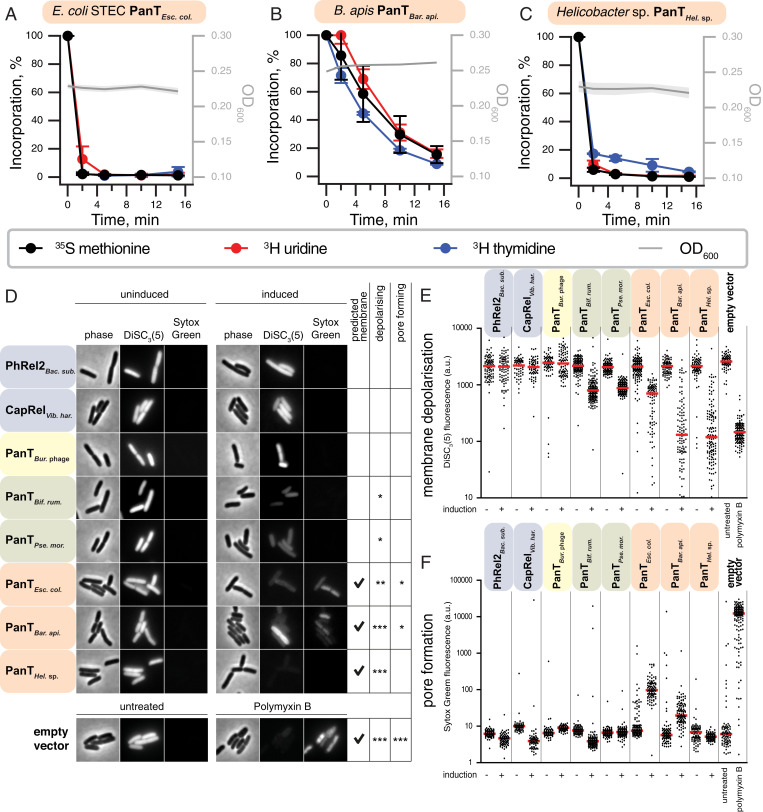
Membrane integrity is a major target of PanT toxins. (*A*–*C*) Metabolic-labeling assays with wild-type *E. coli* BW25113 expressing (*A*) PanT*_Esc. col._*, (*B*) PanT*_Bar. api_*_._, or (*C*) PanT*_Hel._*_sp._ toxins. (*D*) Phase-contrast (*Left*) and fluorescence images (*Middle* and *Right*) of *E. coli* cells costained with membrane potential–sensitive dye DiSC_3_(5) and membrane permeability indicator SYTOX Green. Depicted are representative cells carrying either an empty or PanT-expressing vector under uninducing (no L-arabinose) or inducing (30-min induction with 0.2% L-arabinose) conditions. As a positive control, cells containing empty vector (in MOPS-glucose medium) were incubated for 15 min with membrane-depolarizing and pore-forming antibiotic Polymyxin B. High DiSC_3_(5) fluorescence levels indicate high membrane potential levels found in well-energized, metabolically active cells. High SYTOX Green levels, in contrast, indicate formation of pores in the inner membrane. (*E* and *F*) Quantification of (*E*) DiSC_3_(5) and (*F*) SYTOX Green fluorescence for individual cells from the same imaging dataset (*n* = 92 to 165 cells). Median fluorescence intensity is indicated with a red line. Shading indicates toxin type as per Fig. 2A.

Next, we performed ^35^S methionine, ^3^H uridine, and ^3^H thymidine metabolic labeling experiments with the predicted transmembrane domain harboring toxins PanT*_Esc. col._* (Fig. 5*A*), PanT*_Bar. api._* (Fig. 5*B*), and PanT*_Hel._*_sp._ (Fig. 5*C*). Unlike the toxins of the previous two sections of *Results* that predominantly target translation or transcription, expression of these toxins indiscriminately inhibited transcription, translation, and DNA replication, consistent with a more general shutdown of metabolic activities caused by membrane disruption. Indeed, a comparable response was observed with the induction of membrane-depolarizing *E. coli* HokB TA toxin (35) (*SI Appendix*, Fig. S8*B*) and treatment with the membrane-targeting inhibitor of oxidative phosphorylation carbonyl cyanide 3-chlorophenylhydrazone (*SI Appendix*, Fig. S8*C*).

To directly test this hypothesis, we analyzed the integrity of cell membranes upon toxin induction using a combination of the membrane potential–sensitive dye “DiSC_3_(5)” (36) and inner membrane permeability indicator SYTOX Green (37). A strong membrane depolarization combined with an increased SYTOX Green permeability was observed for PanT*_Bar. api._* and PanT*_Esc. col_*_._ (Fig. 5 *D*–*F*). Expression of PanT*_Hel._*_sp._, in contrast, triggered strong depolarization without an increase in SYTOX Green permeability. Thus, we conclude PanT*_Esc. col._*, PanT*_Hel._*_sp._, and PanT*_Bar. api._* exert their toxic activity through membrane depolarization which, in the case of PanT*_Esc. col._* and PanT*_Bar. api._*, is caused by large pore formation. Finally, weak membrane depolarization was also observed for PanT*_Bif. rum._* and PanT*_Pse. mor._*, although these are not predicted to contain transmembrane helices and are instead predicted to be RNases. Therefore, the effect of these toxins on cell membranes is more likely to be indirect through disturbances in respiration or central carbon metabolism. A potential membrane-spanning region is predicted for PanAT*_Bur. _*_phage_, albeit with relatively weak support (55%) (*SI Appendix*, Fig. S4*D*). As this protein does not appear to affect membrane integrity, its toxicity that is particularly striking in its effect on transcription as described above is more likely to result from its enzymatic activity, putatively cyclic nucleotide synthesis.

### While PanAs Are Naturally Specific for their Cognate PanT Toxins, Their PanT Neutralization Spectrum Can Be Expanded through Directed Evolution.

**Fig. 6.**
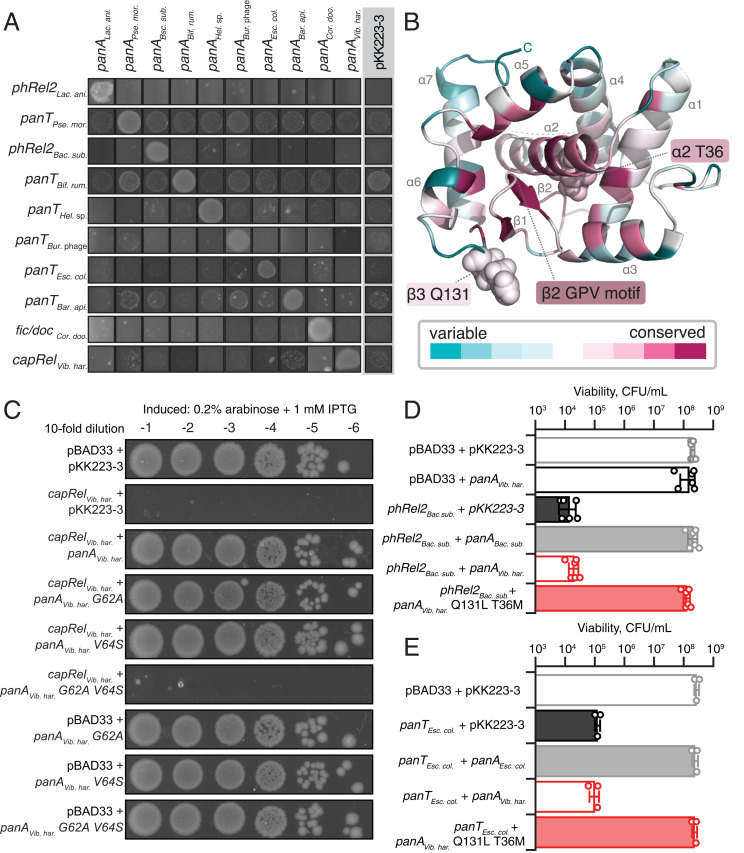
PanA specificity can be readily evolved though directed evolution. (*A*) Exhaustive cross-neutralization testing establishes the strict specificity of PanA antitoxins toward their cognate toxins. The overnight cultures of *E. coli* strains transformed with pBAD33 and pKK223-3 vectors or derivatives thereof expressing toxin and PanA antitoxins was adjusted to 1.0, cultures serially diluted from 10^1^- to 10^6^-fold and spotted on LB agar medium supplemented with appropriate antibiotics as well as inducers (0.2% arabinose for toxin induction and 1 mM IPTG for induction PanA variants); 10^1^-fold dilution is shown. (*B*) trRosetta-predicted structure (TM score 0.70, “very high confidence”) of the PanA*_Vib. har._* antitoxin colored by degree of conservation as per *SI Appendix*, Fig. S11. (*C*) Effect of amino acid substitutions in conserved GPV motif on of PanA*_Vib. har._* antitoxin functionality. (*D*) Neutralization of PhRel2*_Bac. sub._* toxin by evolved noncognate PanA*_Vib. har._* T36M Q131L antitoxin. To quantify the effects of PanA/PanT coexpression on bacterial viability, the overnight cultures were subdiluted and spread on the LB agar, and individual colonies were counted. Analogous experiments with single-substituted T36M and Q131L PanA*_Vib. har._* variants are shown in *SI Appendix*, Fig. S11. (*E*) Neutralization of PanT*_Esc. col_*_._ toxin by wild-type and T36M Q131L PanA*_Vib. har._* variants.

We have earlier shown that Type II antitoxins neutralizing toxSAS toxins—such as *B. subtilis* Ia1a PanA*_Bac. sub._* neutralizing PhRel2*_Bac. sub._*—are specific for their cognate toxins (4). PanA is clearly a versatile domain that can evolve to neutralize—and become specific for—a range of different toxin domains. Therefore, we performed exhaustive cross-inhibition testing, resulting in a 10 × 10 cross-neutralization matrix (Fig. 6*A* and *SI Appendix*, Fig. S10). A clear diagonal signal is indicative of PanA antitoxins naturally efficiently protecting only from cognate toxins—even within groups of evolutionary related toxic effectors such as toxSAS CapRel*_Vib. har._*, PhRel2*_Lac. ani._*, and PhRel2*_Bac. sub._* Conversely, on the evolutionary timescale, Panacea changes its toxin specificity and swaps partners, which raises the questions of what the structurally important regions for neutralization are and how a new specificity profile can be evolved.

The Panacea domain is not identifiably homologous to any protein with a known structure. Therefore, we have de novo predicted the structure of PanA*_Vib. har._* using trRosetta, a deep learning–based method (31) (Fig. 6*B*). The model has a confidence categorized as “very high,” with an estimated template modeling (TM) score of 0.704. Independent structural prediction with AlphaFold2 (10) indicates the same overall fold, with an RMSD of 1.05 Å as calculated by PyMol (*SI Appendix*, Fig. S11). The structure is comprised of a central helix (ɑ2) surrounded by five further helices and a small three-strand β-sheet that contains a strongly conserved GPV amino acid sequence motif in the β2 strand proximal to the central helix ɑ2 (Fig. 6*B* and *SI Appendix*, Fig. S12). The β3 and ɑ2 elements are particularly well conserved in the sequence alignment (*SI Appendix*, Fig. S12). We probed the functional importance of the GPV motif in toxicity neutralization assays. While individual G62A and V64S substitutions did not affect the ability of *panA_Vib. har._* to neutralize its cognate toxin *capRel_Vib. har._*, the G62A V64S double substitution resulted in the loss of neutralization activity (Fig. 6*C*), supporting that the GPV motif is, indeed, functionally important.

Next, we subjected a pair of toxSAS:PanA TA systems with effectors belonging to two distinct toxSAS subfamilies—PhRel2 and CapRel—to directed evolution experiments and screened for mutant variants of PanA*_Vib. har._* that are able to neutralize *B. subtilis* PhRel2*_Bac. sub._* Even though the amino acid identity between PanA*_Vib. har._* and PanA*_Bac. sub._* proteins is only 30 to 40%, just two substitutions—T36M and Q131L—were sufficient for cell viability as judged by colony-counting experiments (Fig. 6*D* and *SI Appendix*, Fig. S13*A*). Individual T36M and Q131L substitutions are not sufficient to elicit cross-reactivity (*SI Appendix*, Fig. S13*A*). T36 is part of the well-conserved central helix ɑ2, while Q131 is located in a small, variable β3 strand. The β2 strand containing the conserved GPV motif is sandwiched between these structural elements (Fig. 6*B*). Notably, the T36M Q131L PanA*_Vib. har._* variant is still capable of protecting from the cognate CapRel*_Vib. har._* toxin. However, the protection from PhRel2*_Bac. sub_*_._ toxicity is less efficient than that conferred by the cognate PanA*_Bac. sub._* antitoxin: the bacterial colonies are smaller, indicative of incomplete detoxification (*SI Appendix*, Fig. S13*A*). Therefore, we hypothesized that the T36M Q131L double substitution does not result in specificity switching in a strict sense but rather relaxes the specificity, thus allowing the neutralization of noncognate toxins. To probe this hypothesis, we tested if T36M Q131L PanA*_Vib. har._* could protect from other noncognate PanTs (Fig. 6*E* and *SI Appendix*, Fig. S13*B*). We found that T36M Q131L PanA*_Vib. har._* can protect from the noncognate cell membrane–targeting PanT*_Esc. col._* (Fig. 6*E*), although incompletely, as evident from the smaller colony size (*SI Appendix*, Fig. S13*B*); no increased protection from other noncognate PanTs was detected (*SI Appendix*, Fig. S13*C*).

## Discussion

**Fig. 7.**
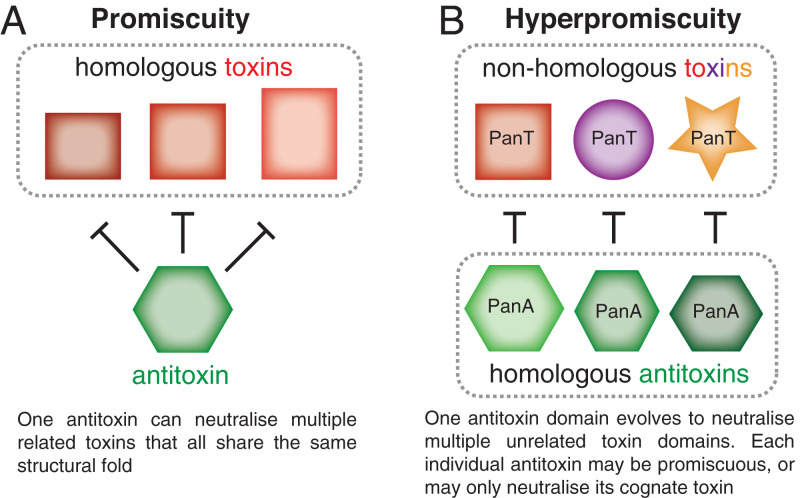
Antitoxin promiscuity versus hyperpromiscuity. (*A*) A promiscuous antitoxin has relaxed neutralization specificity toward its target toxin and can neutralize a range of related toxins which all share the same structural fold. Examples include cross regulation of RelBE-like modules in *Mycobacterium tuberculosis* (55) and promiscuous ParD antitoxins generated through directed evolution that neutralize noncognate ParE toxins (28). (*B*) A hyperpromiscuous antitoxin domain, as exemplified by Panacea, can evolve to neutralize unrelated toxins that share neither structural fold nor mechanism of action.

Type II TAs are highly specific at the sequence level; however, small changes can result in promiscuous intermediates allowing the neutralization of additional homologous but noncognate toxins (28, 38, 39). Through selection experiments, we have demonstrated that via just two amino acid substitutions, Panacea-containing antitoxins can be made to neutralize not just noncognate but *nonhomologous* noncognate toxins that have different cellular targets and mechanisms of action. This reveals a remarkable versatility of the Panacea domain. We suggest describing the ability of an antitoxin domain to evolve to neutralize different toxin domains as *hyperpromiscuity*, distinguishing from *promiscuity*, in which one individual antitoxin can neutralize noncognate but homologous toxins sharing the same structural fold (Fig. 7). A naturally occurring example of the latter can be seen in the bacteriophage T4 antitoxin Dmd that neutralizes the homologous mRNase toxins RnlA and LsoA (26, 27).

Other versatile antitoxin domains have also previously been observed in computational analyses to be associated with multiple toxin-like domains (16, 40, 41), indicating potentially similar plasticity and hyperpromiscuity. One example is the Phd-related antitoxin domain found in proteins that can neutralize RelE-like mRNases, in addition to those that neutralize the EF-Tu phosphorylating toxin Doc (40). DUF4065/Panacea has previously avoided identification as a widespread antitoxin domain, despite its broad distribution in prokaryotic chromosomes. Further bioinformatic investigations of TA systems are required to understand just how unique Panacea is in its hyperpromiscuity and how many more hyperpromiscuous TAs are waiting for discovery in the vast wealth of microbial genomes.

A number of other outstanding questions about PanA remain. Firstly, how is one single domain able to neutralize so many different toxins while insulating itself against noncognate interactions? The answer to this will come from structural analyses of multiple PanAs—both alone and in complex with cognate toxins—combined with additional directed evolution experiments using different PanA pairs and an analysis of sequence coevolution. A structural analysis of complexes will also reveal the molecular function of the conserved GPV motif that is a signature of the Panacea domain and is critical for neutralization. The second question is just how much of a role proteases play in the function of PanA in some species—given the previously observed function of the Panacea domain–containing antitoxin SocA in the proteolytic degradation of toxin SocB in *Caulobacter* (17). While our results are most consistent with a Type II direct mechanism of inactivation rather than the indirect Type VI–like mechanism observed for SocA, the two means of neutralization may not be mutually exclusive. Dual antitoxin functions have previously been observed; for example, DarG of the DarTG system both removes a toxic modification from DNA (Type IV) and directly binds to and inhibits the modifying enzyme DarT (Type II) (42). Finally, the evolutionary forces that drive and enable such ready partner swapping of PanAT pairs are unclear. One answer to this is hinted at in the kinds of proteins that are encoded near PanATs (Dataset S1) and in the analysis of TA (although not PanAT) gene locations near recombination sites of Tn3 transposases (43). We have found that many PanATs are encoded in close enough vicinity to transposase genes for the latter to be predicted as third component TA system genes or even false positive potential toxins that were filtered out by our pipeline (Dataset S1). It is not surprising, nor is it a new observation, that TAs can be associated with transposons; they can potentially act as addiction modules, similar to their role on plasmids (2). It is tempting to speculate that the presence of PanATs near hotspots of genomic rearrangements involving transposons and prophages—combined with an inbuilt versatility of the Panacea domain—could be driving the recombining of pairs that we observe.

## Materials and Methods

### Identification of PanA in Proteomes across the Tree of Life.

From the NCBI genomes index (http://ftp.ncbi.nlm.nih.gov/genomes), we downloaded 20,209 predicted proteomes, selecting all viruses and one representative proteome per species for archaea, bacteria, and eukaryotes. The full taxonomy was also retrieved from NCBI. To detect the presence of PanA across the tree of life, we used the HMM of the DUF4065 domain from the Pfam database (14). We used HMMer v3.1b2 (44) to scan our database of proteomes with the DUF4065 HMM using thresholds set to the HMM profile’s gathering cutoffs. We found that the DUF4065 domain was present in 2,281 identified sequences. We stored the sequences, taxonomy of the source organism, and domain composition in a MySQL database. We used this dataset and subsets of it for further phylogenetic analysis (*SI Appendix*, *Methods*: *Representative sequence dataset assembly* and *Phylogenetic analysis*).

### Prediction of Sequence Features and Structure.

Structural modeling was carried out with the trRosetta server (31). This prediction is based on de novo folding, guided by deep learning restraints. The model was colored by conservation using the Consurf server and an alignment of the sequences shown in Fig. 2 (45). Additional structural prediction was carried out for PanA*_Vib. har._* with the AlphaFold2 (46) Colab notebook with default settings (“advanced” version; https://github.com/sokrypton/ColabFold). RSMD was calculated using structural alignment in PyMol v2.4.2 (pymol.org). Transmembrane regions were predicted with the TMHMM 2.0 sever (default settings). See *SI Appendix*, *Methods*: *Prediction of sequence features and structure* for details of sequence analyses for the prediction of protein domains and the identification of prophage-like genomic regions.

### Prediction of TA Loci.

Our Python tool FlaGs (18), which takes advantage of the sensitive sequence search method Jackhmmer (44), was adapted to identify conserved two- or three-gene conserved architectures that are typical of TA loci. Full details of the method are described in *SI Appendix*, *Methods*: *Prediction of TA loci*, with a schematic of the workflow shown in *SI Appendix*, Fig. S1. All scripts and datasets are available at https://github.com/GCA-VH-lab/Panacea.

### Metabolic Labeling with ^35^S Methionine, ^3^H Uridine, or ^3^H Thymidine.

Metabolic-labeling assays were performed as described previously (4). For details, see *SI Appendix*, *Methods*: *Metabolic labeling*.

### Construction of Plasmids.

All bacterial strains and plasmids used in the study are listed in Dataset S3, and details can be found in *SI Appendix*, *Methods*: *Construction of plasmids*.

### HPLC-Based Nucleotide Quantification.

*E. coli* strain BW2511324 and *E. coli* BW25113 Δ*relA* were transformed with PanT*_Bur._*_phage_–expressing plasmid (pBAD33—*Burkholderia* prophage phi52237) as well as empty pKK223-3 vector. The starter cultures were pregrown overnight at 37 °C with vigorous shaking (200 rpm) in Neidhardt MOPS minimal media supplemented with 1 µg/mL thiamine, 1% glucose, 0.1% caa, 100 µg/mL carbenicillin, and 20 µg/mL chloramphenicol. The overnight cultures were diluted to optical density (OD)_600_ 0.05 in 115 mL prewarmed medium MOPS supplemented with 0.5% glycerol as carbon source and grown until OD_600_ ∼ 0.2 at 37 °C, 200 rpm. At this point, 0.2% arabinose was added to induce the expression of the toxin. A total of 26-mL samples were collected for HPLC analyses at 0, 2, 5, and 10 min after the addition of arabinose and 1 mM IPTG. Nucleotide extraction and HPLC analyses were performed as described previously (34). The OD_600_ measurements were performed in parallel with a collection of the samples for HPLC analyses.

### Toxicity Neutralization Assays.

Toxicity-neutralization assays were performed on Lysogeny broth (LB) medium (Lennox) plates (VWR). *E. coli* BW25113 strains transformed with pBAD33 derivative plasmids encoding toxins [medium copy number, p15A origin of replication, Cml^R^, toxins are expressed under the control of a P_BAD_ promoter (47)] and pKK223-3 derivatives encoding antitoxins [medium copy number, ColE1 origin of replication, Amp^R^, antitoxins are expressed under the control of a P_Tac_ promoter (48)] were grown in liquid LB medium (BD) supplemented with 100 µg/mL carbenicillin (AppliChem) and 20 µg/mL chloramphenicol (AppliChem) as well as 1% glucose (repression conditions). Serial 10-fold dilutions were spotted (5 µL per spot) on solid LB plates containing carbenicillin and chloramphenicol in addition to either 1% glucose (repressive conditions) or 0.2% arabinose combined with 1 mM IPTG (induction conditions). Plates were scored after an overnight incubation at 37 °C.

To quantify bacterial viability (colony forming units, CFU), overnight cultures were diluted to OD_600_ either in the range from 0.1 to 0.01 (for the strains expressing PhRel2*_Bac. sub._*, with and without coexpression of wild-type PanA*_Vib. har._*) or OD_600_ ranging from 1.0 × 10^−4^ to 1.0 × 10^−5^ (all other strains) and spread on the LB agar medium as described above for the spot-test toxicity neutralization assay. The final CFU/mL estimates were normalized to OD_600_ 1.0.

### PanAT Complex Formation.

The plasmids were transformed into the *E. coli* BL21 DE3 strain. Fresh transformants were washed from an LB (BD Difco-Fisher Scientific) agar plate and used to inoculate a 1-L culture LB supplemented with kanamycin (50 µg/mL). The cells were grown on 37 °C until OD_600_ reached 0.4 to 0.5 and induced with 0.5 mM IPTG. The cells were harvested after overnight cultivation on 18 °C, 220 rpm. The cells were opened with sonication in binding buffer (BB: 25 mM Hepes, pH 7.5; 300 mM NaCl; 10 mM imidazole; 2 mM CaCl_2_; 2 mM β-ME). Filtered lysate was incubated with 1 mL previously buffer-equilibrated Ni-beads (His60 Ni Superflow Resin, TaKaRa) for 30 min. Bound protein was washed with BB on a gravity column and eluted with 300 mM imidazole. Fractions were resolved on a 15% sodium dodecyl sulphate–polyacrylamide gel electrophoresis (SDS-PAGE) gel and stained with SimplyBlue SafeStain.

### Fluorescence Microscopy.

Overnight cultures were grown at 37 °C in MOPS minimal medium supplemented with 1% glucose. Next morning, the cells were washed, resuspended, and diluted in MOPS medium supplemented with 0.5% glycerol in order to remove glucose followed by incubation at 37 °C until an OD_600_ of 0.3. For maintaining the plasmids, all cultures were grown in the presence of 34 µg/mL chloramphenicol. Toxin production was induced by the addition of arabinose (0.2%) for 30 min followed by staining with 200 nM of the membrane permeability indicator SYTOX Green (37) alongside the induction and 250 nM membrane potential–sensitive dye DiSC_3_(5) (49) for the last 5 min (36, 50). The samples were immobilized on microscope slides covered with a thin layer of H_2_O/1.2% agarose and imaged immediately. As a positive control for pore formation, BW25113 *E. coli* cells transformed with the empty pBAD33 vector were incubated with 10 µg/mL polymyxin B for 15 min (51). Microscopy was performed using a Nikon Eclipse Ti equipped with a Nikon Plan Apo 100×/1.40 Oil Ph3 objective, CoolLED pE-4000 light source, Photometrics BSI sCMOS camera, and Chroma 49002 (excitation [EX] 470/40, dichroic mirror [DM] 495 lpxr, and emission [EM] 525/50) and Semrock Cy5-4040C (EX 628/40, DM 660 lp, EM 692/40) filter sets. The images were acquired with Metamorph 7.7 (MolecularDevices) and analyzed with Fiji (52).

### Selection of Cross-Inhibiting PanA Mutants.

An error-prone PCR mutant library of *Vibrio Harveyii* PanA antitoxins was created as described in *SI Appendix*, *Methods*: *Selection of cross-neutralizing PanAs: preparation of the antitoxin mutant library*. A total of 5 µL (around 1 µg) antitoxin mutant library was transformed into the BW25113 *E. coli* strain carrying a noncognate toxin expression plasmid PhRel2*_Bac. sub._* toxSAS toxin from *B. subtilis* Ia1a (VHp303). The transformants were let to recover for 1 h in 1 mL SOC media at 37 °C and added to 20 mL LB media supplemented with ampicillin (100 µg/mL), chloramphenicol (25 µg/mL), 0.2% L-arabinose, and 1 mM IPTG. The bacteria were grown overnight at 37 °C while expressing both toxin and antitoxin. Next day, the plasmid was extracted from 3 mL culture using a Favorprep Plasmid Extraction Mini Kit (Favorgen Biotech Corp.). A total of 500 ng plasmid mix was again transformed into BW25113 carrying a toxin expression plasmid and let to recover as before. A total of 100 µL recovery culture was spread on LB agar plates containing corresponding antibiotics as well as 0.2% glucose (control of transformation efficiency), and the rest of the culture was collected by centrifugation and spread on an LB agar plate containing corresponding antibiotics as well as 0.2% L-arabinose and 1 mM IPTG.

Overnight cultures were started from selected colonies for further testing of cross-inhibition. The plasmids were extracted with Favorprep Plasmid Extraction Mini Kit and cleaved with FastDigest *Sac*I restriction enzyme (Thermo Scientific) to eliminate the toxin plasmids. To ensure the purity of the antitoxin mutant mix, it was transformed into the *E. coli* DH5α strain, and the plasmids were extracted from the offspring of a single colony. The *E. coli* BW25113 strain expressing the cognate or noncognate toxin was then transformed with 500 ng mutated plasmid. Again, 100 µL recovery culture was spread onto LB supplemented with corresponding antibiotics as well as 0.2% glucose agar plates, and the rest of the bacteria was collected and spread on LB agar plates supplemented with corresponding antibiotics, 0.2% arabinose and 1 mM IPTG. Phusion High-Fidelity DNA Polymerase (Thermo Scientific) was used to amplify the *panA* mutant (pK223_fwd_CPEC and pK223_rev_CPEC primers) and toxin genes (pBAD_fwd and pBAD_rev primers) with colony PCR and sequenced using pK223_rev_CPEC or pBAD_fwd primer correspondingly. The plasmid mixes and bacterial colonies were tested for possible contamination at various steps using FIREPol DNA Polymerase (Solis BioDyne): antitoxins were tested with the combination of pK223_rev_CPEC and STEC_panA_ctrl2, VH_panA_ctrl1, or Bsup_panA_ctrl1 primers and toxins with the combination of pBAD_fwd and STEC_TOX_ctrl1, VH_TOX_ctrl1, and Bsup_TOX_ctrl1 (Dataset S3).

## Supplementary Material

Supplementary File

Supplementary File

Supplementary File

Supplementary File

## Data Availability

Python code and text files of alignments, trees, and HMMs have been deposited in GitHub (https://github.com/GCA-VH-lab/Panacea) (56). All other study data are included in the article and/or supporting information.
