# Supplementary figures and images for: A hyperpromiscuous antitoxin protein domain for the neutralization of diverse toxin domains
Source: Proc Natl Acad Sci U S A. 2022 Feb 4;119(6):e2102212119. doi: 10.1073/pnas.2102212119 (PMC8832971; doi:10.1073/pnas.2102212119)

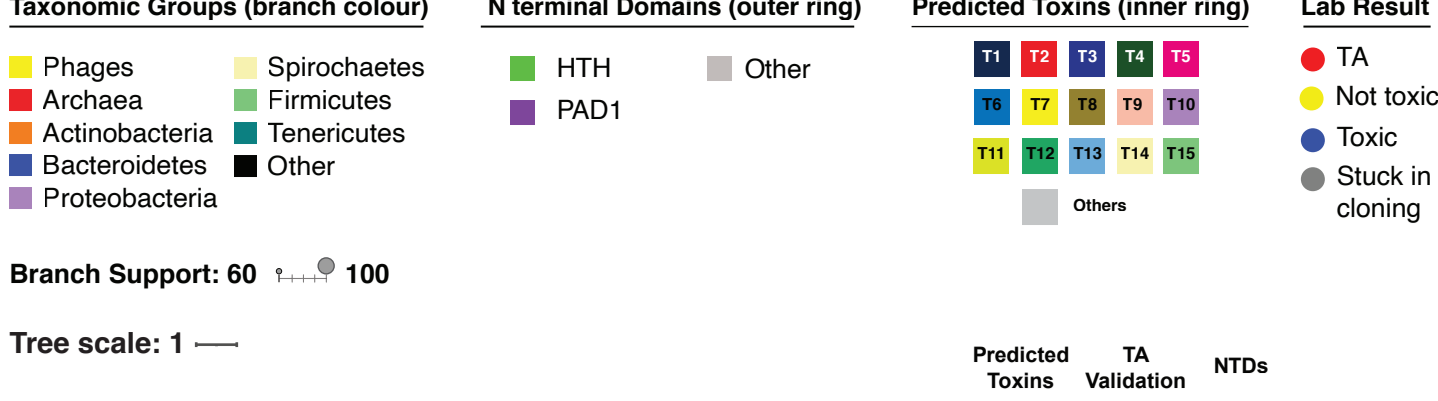

Branch Support: 60 100

Tree scale: 1 —

Predicted Toxins    TA Validation    NTDS

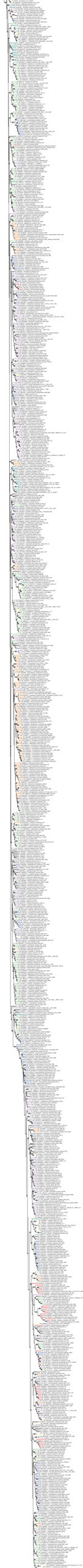

Supplement: Supplementary File [file pnas.2102212119.sd02.pdf]
